# A deep hybrid learning framework with attention-enhanced feature extraction for BMI prediction based on physical fitness

**DOI:** 10.3389/fpubh.2025.1640226

**Published:** 2025-09-02

**Authors:** Ming Mo, Wanhong Luo, Qiao Hu, Jun Wang, Tianshuo Jiao, Libo Xie, Guixiang Wu, Ye Yang, Jinfeng Deng, Xuyin Xu

**Affiliations:** ^1^Changsha Aeronautical Vocational and Technical College, Changsha, Hunan, China; ^2^Hunan First Normal University, Changsha, Hunan, China; ^3^Hunan University, Changsha, Hunan, China; ^4^Hunan Wuxu Network Technology Co., Ltd., Changsha, Hunan, China; ^5^Hunan Yanpei Technical School, Changsha, Hunan, China

**Keywords:** physical fitness, BMI classification, machine learning, attention mechanism, LightGBM, CNN1D hybrid model, university student health monitoring

## Abstract

**Background:**

Body Mass Index (BMI) assessment remains a critical challenge in university health monitoring, where traditional methods fail to capture dynamic relationships between physical fitness indicators and body composition. This study develops a novel predictive framework to address this gap through advanced machine learning techniques applied to longitudinal fitness data from Chinese university students.

**Methods:**

We analyzed 6,698 male students' fitness records (2018–2022) using a hybrid CNN1D-Attention-LightGBM architecture. The model integrates temporal pattern recognition via sliding windows, multi-kernel convolutional operations for physiological coupling analysis, and dynamic attention weighting. Performance was validated through 10-fold cross-validation against SVM and XGBoost benchmarks.

**Results:**

The model achieved 94.5% accuracy (F1 = 0.93), significantly outperforming conventional methods (XGBoost: 90.1%). Cardiorespiratory endurance (3000 m run, r = 0.2009) and upper-body strength (pull-ups, r = −0.2786) emerged as primary BMI determinants. The framework successfully classified four BMI categories (normal weight: 4,991; obese: 82) .

**Conclusion:**

This study establishes the first unified solution for fitness-informed BMI prediction, though limited by male-only sampling. Implementation should prioritize integration with campus health systems and expansion to diverse populations. Future work should incorporate psychosocial factors and multi-regional validation.

## 1 Introduction

In contemporary society, the health status of university students has garnered increasing attention, as physical wellbeing serves as a cornerstone for both academic success and long-term quality of life. Although physical fitness may indirectly influence academic performance through interactions with various factors such as cognitive abilities, mental health, and socioeconomic background, it remains a critical determinant of a healthy lifestyle. Physical fitness, which encompasses components such as cardiorespiratory function, muscular strength, power, and endurance, is closely linked to overall health and can be enhanced through targeted training methods (1–4). Experts in education and public health consistently advocate strengthening students' physical fitness to improve their overall health levels. To comprehensively evaluate students' performance across key fitness domains, this study selected four representative assessments: the 3,000-meter run, 30 × 2 shuttle run, pull-ups, and sit-ups.

Body Mass Index (BMI), a widely used metric for assessing health status, reflects the relationship between body mass and height, though it does not comprehensively capture body composition or overall health. This study transcends simple BMI calculation by establishing a predictive-prescriptive paradigm grounded in the established association between BMI and athletic performance. This paradigm enables large-scale predictive management through projecting BMI trends under target fitness scenarios. It facilitates goal-driven optimization to infer BMI categories where direct measurement is impractical and supports resource prioritization by identifying students exhibiting diverging fitness-BMI trajectories for targeted interventions. Critically, research demonstrates that the relationship between BMI and performance exhibits a distance-dependent pattern (5). While higher BMI correlates with enhanced performance in certain athletic disciplines, endurance events distinctly require optimization toward lower BMI values for peak outcomes. This pattern of distance-specific BMI optimization confirms the necessity of tailored predictive models within the proposed framework.

Recent studies have increasingly applied machine learning and deep learning models–such as decision trees, support vector machines, gradient boosting, and convolutional neural networks (CNNs)—to classify BMI based on fitness indicators (6–10). Innovations such as attention mechanisms have further improved predictive performance by emphasizing relevant features (11, 12). Hybrid architectures, including CNNs combined with wavelet transforms or other deep models, have demonstrated strong capabilities for modeling complex health data (13–15), while LightGBM has been widely adopted for efficient classification in fitness-related tasks (16–18). Class imbalance–a common issue in real-world health data–has been addressed using techniques such as SMOTE or cost-sensitive learning (19, 20). The model incorporates a weighted cross-entropy loss function to enforce balanced learning for all BMI categories.

Although previous research has demonstrated the utility of individual machine learning techniques, most approaches rely on static or handcrafted features, overlooking two critical data structures inherent in longitudinal fitness assessments: temporal structure, referring to time-dependent patterns in sequential measurements, and interrelated structure, representing physiological couplings between metrics. Our CNN1D-Attention-LightGBM framework directly addresses these gaps by employing sliding window segmentation to capture temporal patterns and utilizing multi-kernel convolution to model complex feature interactions.

This hybrid design enables the first unified solution for fitness-BMI dynamics prediction, shifting from passive measurement to proactive health planning.

## 2 Materials and methods

### 2.1 Physical fitness test protocol

This study received ethical exemption for retrospective analysis of pre-existing anonymized data, as certified by the Human Research Ethics Committee of Changsha Aeronautical Vocational and Technical College (hereafter “the Ethics Committee”). The exemption complied with China's Regulations on Ethical Review of Life Sciences and Medicine Involving Humans through:

**Minimal-risk data sourcing:** Anonymized routine fitness records (2018-2022) requiring no participant contact, physiological intervention, or privacy-compromising processes;

**Institutional data legitimacy:** Exclusively institutional physical test archives acquired per educational administration protocols without disrupting teaching activities;

**Retrospective design adherence:** Historical data analysis excluding prospective collection or behavioral interventions.

Consequently, informed consent was waived under ethical exemption standards. A two-stage anonymization protocol was implemented: (i) Removing direct identifiers (names/student IDs) during collection; (ii) Applying numerical perturbation to anthropometrics (±0.5 cm height; ±1 kg weight) to prevent re-identification.

This study employed four key fitness tests to assess the physical fitness of university students, focusing on cardiorespiratory endurance, muscular strength, core endurance, speed, and agility. These tests included the 3,000-meter run, pull-ups, sit-ups, and the 30-meter x2 shuttle run. The following is a detailed description of each test.

#### 2.1.1 3,000-meter run

The 3,000-meter run is primarily used to assess students cardiorespiratory endurance, which reflects the ability of the cardiovascular and respiratory systems to support physical activity over extended periods.

**Test method**: Participants are required to run 3,000 meters (7.5 laps) on a standard 400-meter track. The test is usually conducted in groups, and students are instructed to complete the run as quickly as possible, with their time recorded. Students are advised to maintain a steady pace to avoid excessive fatigue or stopping mid-race.

**Evaluation criteria**: The time required to complete the 3,000 meters is the key indicator of cardiorespiratory endurance. The passing standard for male university students is typically 13 minutes and 30 seconds. Students exceeding this time may exhibit weaker cardiorespiratory endurance.

#### 2.1.2 Pull-ups

Pull-ups are an important test of upper body muscular strength and endurance, particularly targeting the latissimus dorsi, biceps, and deltoid muscles.

**Test method**: Participants perform as many valid pull-ups as possible on a horizontal bar. The test begins in a full-hang position, with students required to pull their body up until their chin surpasses the bar, then return to the hanging position. This is repeated until they can no longer perform a standard pull-up or choose to stop.

**Evaluation criteria**: The number of standard pull-ups completed is recorded. Students with stronger upper body strength typically perform more pull-ups, with 10 repetitions generally considered the passing standard. Those unable to meet this standard may demonstrate insufficient upper body strength.

#### 2.1.3 Sit-ups

The sit-up test primarily evaluates core muscular endurance, particularly the strength and endurance of the abdominal muscles.

**Test method**: Participants lie flat on a mat with knees bent and feet flat, which are held in place by an assistant. Arms are crossed over the chest. Within one minute, participants perform as many sit-ups as possible, ensuring that each movement raises the torso until elbows touch the knees, followed by a return to the fully lying position.

**Evaluation criteria**: The number of standard sit-ups completed in one minute is recorded. Students with strong core endurance typically complete more repetitions, with 40 repetitions considered the passing standard. Those unable to meet this standard may exhibit insufficient core endurance.

#### 2.1.4 30-meter x2 shuttle run

The 30-meter x2 shuttle run assesses speed, agility, and coordination, particularly focusing on rapid starts and directional changes.

**Test method**: The test is conducted on a 30-m marked track. Students start at the baseline, sprint to the opposite marker, touch it, and return to the starting point, completing two round trips for a total of 60 m. The goal is to complete the run as quickly as possible, with the time recorded using a stopwatch.

**Evaluation criteria**: The time to complete the 60-m shuttle run is the key measure. For male university students, the passing standard is generally under 10 s. Students failing to meet this time may show poor sprinting speed and agility.

These four tests evaluate different aspects of physical fitness:

- 3,000-Meter Run: Assesses cardiorespiratory endurance, helping to understand students performance in prolonged physical activity.
- Pull-ups: Focuses on upper body strength and endurance, particularly shoulder and back muscles.
- Sit-ups: Evaluates core endurance, crucial for maintaining proper posture and preventing injuries.
- 30-m x2 shuttle run: Tests speed and agility, reflecting students explosive power and quick directional changes.

The results of these tests provide a comprehensive assessment of students' fitness levels and offer important reference points for health interventions and physical education. The assessment was conducted over four years, starting in September 2018 and concluding in December 2022. The test protocol aimed to fully evaluate students' cardiorespiratory endurance, muscular strength, speed and agility, flexibility, and coordination, covering key aspects of physical fitness and reflecting individual overall motor competence.

To ensure the scientific validity and reliability of the results, all tests were administered by two professionally trained physical education coaches and one medical officer following standardized procedures. The medical officer was specifically responsible for real-time health monitoring and emergency response, while coaches executed test protocols.

External conditions were carefully controlled to ensure data comparability: Indoor temperature was rigorously maintained at 20 ± 2 °C, while relative humidity levels were controlled below 60 percent using centralized HVAC systems. Environmental parameters were recorded at 10-minute intervals throughout testing sessions by HT-800 sensors (±0.5 °C accuracy for temperature; ±3% RH accuracy for humidity).

Measurement instrumentation included: Electronic weight scales (Seca 874, ±0.1 kg accuracy) calibrated quarterly; Laser timing gates (Brower TC-System, ±0.01 s accuracy) for speed tests; Digitized tablets (iPad 9th Gen) running custom software for direct data entry into encrypted SQL databases.

Participants were required to wear appropriate athletic clothing and footwear to perform the tests under optimal conditions. Each participant completed a five-minute warm-up before the formal test to activate body functions and maximize performance. If a participant experienced discomfort during any test, the test was immediately stopped to ensure their safety. An eight-minute rest period was scheduled between tests to avoid fatigue affecting results. To ensure accuracy and repeatability, each test was conducted twice under the same conditions, with a three-month interval between tests.

Anthropometric measurements, including height (cm) and weight (kg), were recorded with participants wearing suitable athletic shoes and lightweight clothing. The researchers then calculated each participant body mass index (BMI), a commonly used metric for assessing obesity.

This large-scale, systematic physical fitness assessment targets gaining a comprehensive understanding of the fitness levels of Chinese university students and identifying potential factors influencing their physical performance. It provides data support for optimizing university physical education and establishes the foundation for personalized fitness training programs. Furthermore, the results deliver scientific evidence for future health promotion activities, enabling the formulation of effective fitness improvement strategies and overall health enhancement for university students.

In addition to regular fitness assessments, future research will explore the interplay between fitness and lifestyle habits, health status, and psychological factors. This comprehensive perspective will contribute to a more complete understanding of the development and influencing factors of university students' physical fitness, offering targeted recommendations and interventions to enhance fitness and promote healthy lifestyles.

### 2.2 Medical examination

The medical examination consisted of three parts: a health screening questionnaire, medical history evaluation, and a physical examination.

A questionnaire was designed for this study, as shown in Table 1, to assess the participants' medical history and exclude individuals who did not meet the research criteria.

**Table 1:** Health status questionnaire.

**No**.	**Question**	**Options**
1	What is your age (years)?	□ 18-20 □ 21-25 □ 26 and above
2	What is your height (cm)?	–––––––
3	What is your weight (kg)?	–––––––
4	Do you have any medical history (if any)?	□ Hypertension □ Diabetes □ Heart disease □ Asthma □ Bone or joint disease □ Other: –––––––
5	Are you currently taking any medication?	□ Yes □ No If yes, please list the medication and its purpose: –––––––
6	What is your exercise habit?	□ Regular exercise □ Occasional exercise □ No exercise

The physical examinations were conducted at the university hospital and included the following assessments: functional examination, internal medicine examination, surgical examination, ear-nose-throat (ENT) examination, and blood tests, including lipid profile, blood glucose, and renal function tests. Vital sign measurements included blood pressure and pulse rate. Cardiac function was assessed using electrocardiography (ECG) to record heart rate, rhythm, and electrical activity. Additional tests were conducted as needed, following standard hospital protocols.

The final test results were submitted to medical experts for evaluation, and participants suitability for the rigorous physical fitness tests was determined based on the Physical Activity Readiness Questionnaire (PAR-Q) issued jointly by the American Heart Association (AHA) and the American College of Sports Medicine (ACSM). The evaluation process included the questionnaire, physical examination, and a comprehensive assessment by medical experts. During the evaluation, the experts carefully analyzed the participants' responses to the questionnaire, with a focus on their medical history, recent health status, and the presence of any cardiovascular, respiratory, or other conditions that could affect physical performance. The goal was to ensure that participants were in good health and free from any significant abnormalities before undergoing the fitness tests.

This process not only helped protect the participants' health and prevent unexpected incidents during testing but also established a solid foundation for the subsequent fitness tests, thereby improving the accuracy and reliability of the test results.

All medical records were stored on password-encrypted servers within the university's Health Promotion Center, with access restricted to two authorized medical staff. These data served exclusively for pre-test safety screening to exclude participants with contraindications (e.g., cardiovascular abnormalities), following AHA/ACSM protocols. No medical data were used as model inputs or features in the machine learning framework.

Raw medical records were archived for 3 years post-study completion, while anonymized fitness data were retained indefinitely for research replication.

### 2.3 Experimental model architecture and data mining framework

The relationship between motor competence-related physical fitness (MCPF) and BMI was transformed into a classification problem in this study. The BMI values of the participants were classified into four categories according to the standards of the World Health Organization (WHO): underweight (A), normal weight (B), overweight (C), and obesity (D). A hybrid model combining convolutional neural networks (CNN) with an attention mechanism and a gradient boosting decision tree (LightGBM) was proposed to explore the relationship between motor competence-related physical fitness (MCPF) and BMI. Convolutional neural networks (CNN) are deep learning models particularly suited for processing grid-structured data. CNNs effectively extract spatial features through local connections, weight sharing, and pooling layers. The attention mechanism is a deep learning technique that enables the model to dynamically focus on the most important parts of the input data by assigning higher weights to features relevant to the task at hand. LightGBM is an efficient gradient boosting decision tree (GBDT) algorithm known for its fast training and strong classification capabilities. The hybrid model leverages the feature extraction power of CNN, with the attention mechanism further enhancing focus on key features, thereby improving the model's generalization. LightGBM then takes these extracted features, applying decision tree-based learning to optimize classification performance. By combining deep learning with gradient boosting, this model captures complex non-linear relationships in the data, significantly improving classification accuracy and efficiency.

Let *x*_*i*_ represent the physical fitness test results of the i-th participant. As mentioned, BMI values are classified into four categories: underweight (A), normal weight (B), overweight (C), and obesity (D). Let *y*_*i*_∈{*A, B, C, D*} represent the BMI classification of the i-th participant. The relationship between the fitness test results and the BMI classification of the i-th participant is represented as (*x*_*i*_, *y*_*i*_). The aim of this study is to use machine learning models to explore the association between the physical fitness test results and BMI classification.

The collected data from the participants were processed into a dataset *D* = {(*x*_*i*_, *y*_*i*_)}, where $x_{i}\in {\mathbb{R}}^{n}$ represents the feature vector of the *i*-th participant, *y*_*i*_∈ℝ represents the corresponding label or output, and *n* is the dimension of the feature space. First, the data were trained using a one-dimensional convolutional neural network (CNN1D) with an attention mechanism.


(1) $$\begin{array}{l} {ŷ}_{i}^{\text{CNN}}=f_{\text{CNN}}\left(x_{i};W_{\text{CNN}}\right) \end{array}$$

where *f*_CNN_(·) is the forward propagation function of the CNN1D model, and *W*_CNN_ represents the model parameters. After introducing the attention mechanism, the weighted feature output of the model is:


(2) $$\begin{array}{l} {ŷ}_{i}^{\text{Attention}}=f_{\text{Attention}}\left({ŷ}_{i}^{\text{CNN}},\alpha_{i}\right) \end{array}$$

where α_*i*_ is the attention weight, and *f*_Attention_(·) represents the feature representation after applying the attention mechanism. CNN1D effectively captures local features in time-series data, making it particularly suited for processing the sequential indicators in physical fitness tests. The introduction of the attention mechanism augmented the model's feature extraction capabilities, facilitating global identification of key features for BMI prediction.

The learned feature weights from the CNN1D-Attention model were then imported into the LightGBM model, where the feature weights are represented as *W*_CNN_. In the LightGBM model, the input features are:


(3) $$\begin{array}{l} {\tilde{x}}_{i}=f_{\text{Attention}}\left(x_{i};W_{\text{CNN}}\right) \end{array}$$

The output of the LightGBM model is:


(4) $$\begin{array}{l} {ŷ}_{i}^{\text{LightGBM}}=\overset{K}{\underset{k=1}{\sum }}f_{k}\left({\tilde{x}}_{i};W_{\text{LightGBM}}\right) \end{array}$$

where *f*_*k*_(·) is the prediction function of the *k*-th decision tree, *W*_LightGBM_ represents the parameters of the LightGBM model, and *K* is the total number of trees. During training, the model's loss function *L*is minimized, defined as:


(5) $$\begin{array}{l} L=\overset{N}{\underset{i=1}{\sum }}l\left(y_{i},{ŷ}_{i}^{\text{LightGBM}}\right)+\Omega \left(W_{\text{LightGBM}}\right) \end{array}$$

where *l*(·) is the loss function (e.g., mean squared error or cross-entropy), Ω(·) is the regularization term to control model complexity, and *N* is the number of participants. The final output of the model is:


(6) $$\begin{array}{l} {ŷ}_{i}={ŷ}_{i}^{\text{LightGBM}} \end{array}$$

This output represents the predicted BMI category for each participant.

Specifically, the feature weights learned by the CNN1D-Attention model are used as initial features for LightGBM, which helps the LightGBM model converge faster and further improves prediction performance. In this step, LightGBM is retrained on the dataset using the initial feature weights to generate the final model.

In this study, handling imbalanced datasets is a key challenge in model design. Due to the uneven distribution of sample sizes across different BMI categories, the model may tend to favor the majority class (e.g., normal weight) while neglecting minority classes (e.g., underweight or obesity). To ensure accurate classification across all categories, a weighted cross-entropy loss function was introduced to address class imbalance in the dataset.

The cross-entropy loss function, commonly used in classification tasks, measures the difference between the predicted and true class distributions. To address class imbalance across BMI categories, differential class weights were assigned to enhance classification accuracy for minority samples. The cross-entropy loss function is defined as:


(7) $$\begin{array}{l} \text{CrossEntropyLoss}=-\overset{N}{\underset{i=1}{\sum }}w_{i}\cdot y_{i}\cdot \log\left(p_{i}\right) \end{array}$$

where *w*_*i*_ is the weight of class *i*, *y*_*i*_ is the true label, and *p*_*i*_ is the predicted probability. In this formula, the weight *w*_*i*_ is calculated as the inverse of the sample size for each BMI category:


(8) $$\begin{array}{l} w_{i}=\frac{1}{\text{class\_count}\left(i\right)} \end{array}$$

where class_count(*i*) is the number of samples in class *i*.

This weight adjustment increases the contribution of minority samples to the overall loss, balancing the model's attention across different categories and addressing the class imbalance problem. The main goal of handling imbalanced datasets is to prevent the model from favoring the majority class during training and to improve prediction accuracy for minority classes. For the BMI classification problem, underweight (A) and obesity (D) categories have fewer samples, but they are of significant importance in health assessments. The application of a weighted cross-entropy loss ensures model focus on minority classes, preventing their oversight.

The class weights are set based on the sample size of each category in the dataset. The four BMI categories are defined as: underweight (A), normal weight (B), overweight (C), and obesity (D), with corresponding sample sizes *N*_*A*_, *N*_*B*_, *N*_*C*_, *N*_*D*_. The class weights can be calculated as:


(9) $$\begin{array}{l} w_{A}=\frac{1}{N_{A}},\;w_{B}=\frac{1}{N_{B}},\;w_{C}=\frac{1}{N_{C}},\;w_{D}=\frac{1}{N_{D}} \end{array}$$

In this way, the model increases the contribution of minority samples to the loss function, improving its ability to predict minority classes.

This study involves four main fitness tasks: the 3,000-meter run, pull-ups, sit-ups, and the 30-meter x2 shuttle run. To incorporate task-specific impacts during training, a multi-task weighted loss function was implemented, assigning differential weights to each fitness task for calibrated influence on predictions. For the four fitness tasks–3,000-meter run, pull-ups, sit-ups, and 30-meter x2 shuttle run–the loss function is defined as:


(10) $$\begin{array}{l} L_{\text{multi-task}}=\alpha \cdot L_{\text{3,000 m}}+\beta \cdot L_{\text{pull-ups}}+\gamma \cdot L_{\text{sit-ups}}+\delta \cdot L_{\text{30*2}} \end{array}$$

where:

- *L*_3000m_: loss for the 3,000-meter run (mean squared error),

- *L*_pull-ups_: loss for pull-ups (cross-entropy loss),

- *L*_sit-ups_: loss for sit-ups (cross-entropy loss),

- *L*_30*2_: loss for the 30-meter x2 shuttle run (mean squared error).

The weights (α, β, γ, δ) are assigned based on both empirical correlation analysis and domain knowledge of physical test relevance. The 3,000 m run exhibits the highest positive correlation with BMI (0.2009), justifying a higher weight α = 0.4. While pull-ups show a strong negative correlation (-0.2786), their weight β = 0.2 is kept consistent with the other strength-related tasks to maintain balance across task types. Sit-ups and the 30 × 2 shuttle run, with weaker correlations, are also assigned weights of 0.2. These values were manually tuned in this study, and future work will explore learnable or adaptive weight strategies.

The attention mechanism enhances the model's feature extraction ability by assigning different weights to different features. To incorporate the attention mechanism's impact into the loss function, an attention loss term was introduced:


(11) $$\begin{array}{l} \begin{array}{c} L_{\text{attention}}=\lambda \cdot \overset{N}{\underset{i=1}{\sum }}\text{AttentionWeights}\cdot \\ \text{CrossEntropyLoss}\left(\text{outputs},\text{targets}\right) \end{array} \end{array}$$

where λ is the coefficient that controls the weight of the attention loss, balancing the influence of the attention mechanism.

**Figure 1:**
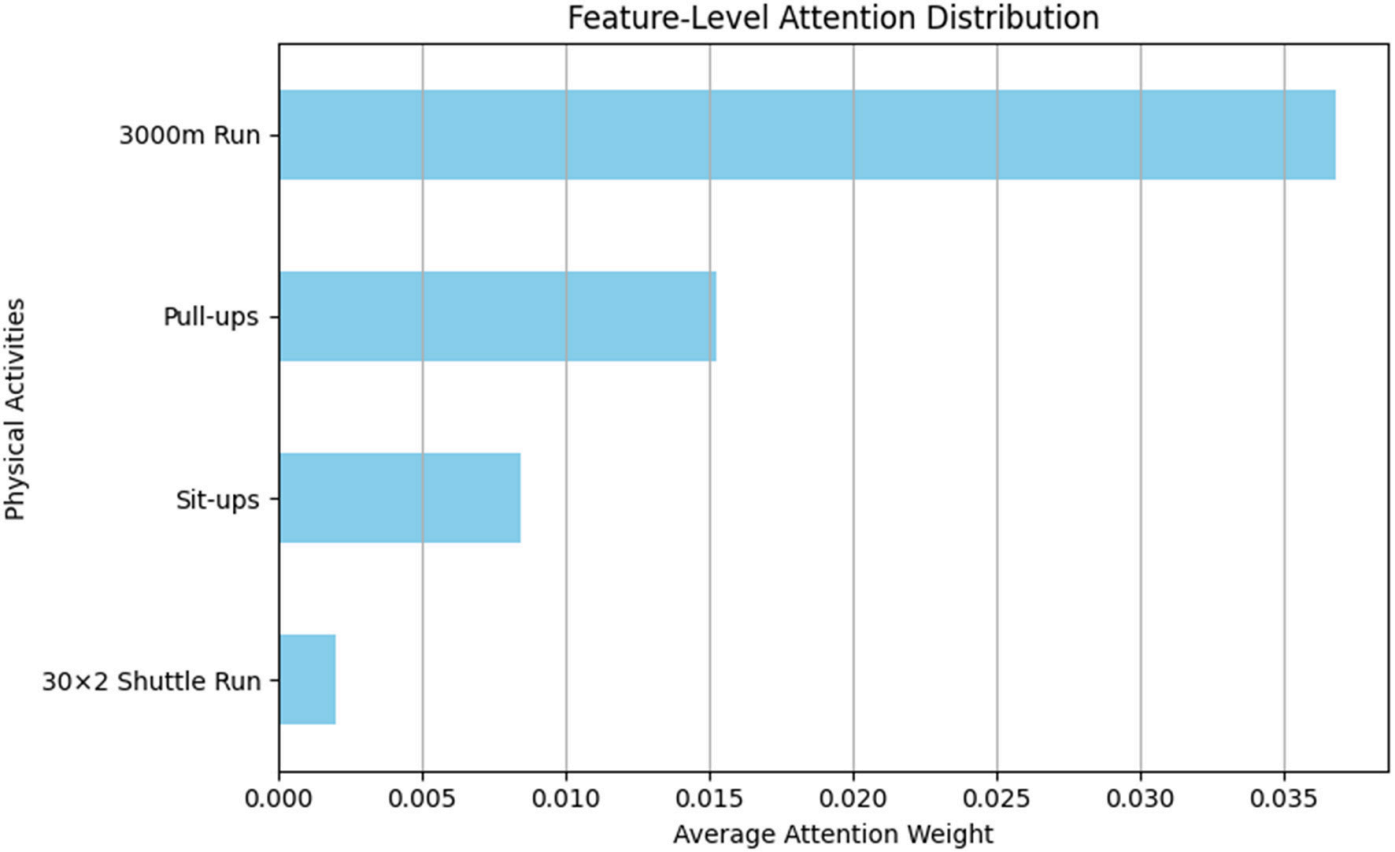
Average attention weight assigned to each physical test feature by the CNN-Attention module.

This loss function ensures that the model dynamically adjusts the weight distribution across different features, yielding more accurate predictions. Furthermore, the attention weights generated during training allow us to interpret the importance of each input feature. As shown in Figure 1, the model assigns the highest average attention to the 3,000 m run, which aligns with the strongest empirical correlations with BMI observed in Table 2. This consistency between data-driven correlation analysis and attention-based feature weighting demonstrates the interpretability of our hybrid model.

**Table 2:** Correlation coefficients between physical fitness tests and BMI classification.

**Assessment metrics**	**BMI**	**3,000 m**	**Pull-ups**	**Sit-ups**	**30 × 2**
BMI	-	0.200954	-0.278618	-0.045984	0.005206
3,000 m	0.200954	-	-0.405648	-0.131292	0.007651
Pull-ups	-0.278618	-0.405648	-	0.207525	-0.010552
Sit-ups	-0.045984	-0.131292	0.207525	-	-0.01116
30x2	0.005206	0.007651	-0.010552	-0.01116	-

This loss function ensures that the model dynamically adjusts the weight distribution across different features, yielding more accurate predictions.

Combining the above loss terms, the total loss function for this study is expressed as:


(12) $$\begin{array}{c} L_{\text{total}}=L_{\text{multi-task}}+L_{\text{attention}}+L_{\text{class-weighted CE}} \end{array}$$

where *L*_multi-task_ is the weighted loss for the four fitness tasks, *L*_attention_ is the loss for the attention mechanism, and *L*_class-weighted CE_ is the weighted cross-entropy loss for addressing class imbalance. This combined loss function effectively resolves the class imbalance problem while fully leveraging multi-task learning and the attention mechanism to improve the model's prediction performance and interpretability.

## 3 Results

The results of the participants' motor competence-related physical fitness tests and BMI classifications are shown in Table 3. The results indicate that 355 male university students were classified as underweight, 4,991 as normal weight, 1,270 as overweight, and 82 as obese.

**Table 3:** BMI distribution of male university students in Central China.

**Weight status**	**Male**
Underweight	355
Normal weight	4,991
Overweight	1,270
Obesity	82

### 3.1 Design decision

The design decisions in this study were based on an in-depth exploration of the relationship between motor competence-related physical fitness (MCPF) and BMI classification. Convolutional neural networks (CNN) were integrated with an attention mechanism and a gradient boosting decision tree (LightGBM), leveraging deep learning-based feature extraction alongside traditional machine learning classification capabilities. The specific design decisions are as follows:

#### 3.1.1 Data processing and feature extraction

The motor competence-related physical fitness test results and BMI data collected from university students in Central China were first preprocessed. Data normalization ensured all features occupied the same numerical range. Subsequently, a one-dimensional convolutional neural network (CNN1D) processed the physical fitness test data to extract local features.

#### 3.1.2 Incorporation of attention mechanism

Given the varying importance of fitness data features for BMI classification, an attention mechanism was incorporated into the CNN architecture. This mechanism assigns differential weights to features, prioritizing those most relevant to BMI classification and enhancing the model's discriminative ability.

#### 3.1.3 Selection of hybrid model

To enhance classification performance and model generalizability, a hybrid architecture was adopted. First, the CNN1D-Attention model was used to perform initial training on the data, and the learned feature weights were fed into the LightGBM model. LightGBM, as an efficient gradient boosting decision tree algorithm, provides fast training and effectively handles complex relationships between features. This design not only accelerated model convergence but also improved prediction accuracy and robustness.

#### 3.1.4 Data splitting and evaluation metrics

The dataset was split into 70% for training and 30% for testing. Accuracy and F1 score were adopted as primary evaluation metrics for model performance assessment. Accuracy measures the overall correctness of the model and is calculated as:


(13) $$\begin{array}{c} \text{accuracy}=\frac{TP+TN}{TP+TN+FP+FN} \end{array}$$

where TP represents true positives, TN true negatives, FP false positives, and FN false negatives.

The F1 score combines precision and recall, providing a more balanced performance evaluation. It is calculated as:


(14) $$\begin{array}{c} F_{\alpha }=\frac{\left(1+\alpha^{2}\right)TP}{\left(1+\alpha^{2}\right)TP+\alpha^{2}FN+FP} \end{array}$$

Precision and recall were weighted equally in this study, with α consequently set to 1. It should be noted that the best *F*_α_ score is 1, while the worst is 0.

The dataset used in this study included four motor competence-related physical fitness tests (3,000-meter run, pull-ups, sit-ups, and 30x2 shuttle run) along with the corresponding BMI classifications (BMI_Category). Data analysis and model training were employed to explore the complex relationship between motor competence-related physical fitness (MCPF) and BMI classification, with predictions generated via the hybrid model.

Table 3 shows that cardiorespiratory endurance tests (3,000 m: 0.2009; 30 × 2 shuttle: 0.0052) positively correlate with BMI, indicating that individuals with higher BMI perform worse in endurance tasks (21). Strength tests (pull-ups: -0.2786; sit-ups: -0.0459) are negatively correlated, especially pull-ups, suggesting higher BMI impairs strength.

The CNN1D-Attention+LightGBM hybrid model identified the 3,000 m run as having the greatest impact on BMI prediction, with sit-ups demonstrating lower contribution. The hybrid outperformed SVM and XGBoost on accuracy and F1, accurately distinguishing normal vs. overweight categories, confirming that fitness data reliably reflect BMI differences.

Overall, cardiorespiratory endurance (3,000 m run) and upper-body strength (pull-ups) are key BMI predictors. The proposed model achieved excellent performance, demonstrating that deep-learning-extracted features from these tests can accurately classify BMI categories and support targeted health interventions for university students.

## 4 Discussion

### 4.1 Model training

Comparative experiments evaluated the proposed CNN1D-Attention-LightGBM hybrid model against baseline classifiers: Naive Bayes (NB), Support Vector Machine (SVM), Artificial Neural Network (ANN), and XGBoost. Table 4 presents the accuracy and F1 score comparisons of these models.

**Table 4:** Comparison of accuracy and F1 scores between the proposed framework and other classifiers.

**Algorithm model**	**Accuracy**	**F1 score**
NB	75.40%	0.72
SVM	82.30%	0.80
ANN	88.60%	0.87
XGBoost	90.10%	0.89
CNN1D-Attention	91.40%	0.90
CNN1D-Attention+LightGBM	**94.50%**	**0.93**

Bold values indicate the optimal performance metrics among comparative methods.

In the experiments, Naive Bayes (NB), as a basic probabilistic model that assumes feature independence, performed less effectively on BMI classification tasks. Although NB is computationally efficient, its performance is limited when dealing with complex, high-dimensional data. As a result, the NB model showed lower accuracy and F1 scores compared to other models.

Support Vector Machine (SVM), which seeks the optimal hyperplane in high-dimensional space, performed better than Naive Bayes in handling complex feature data. However, while SVM achieved higher accuracy and F1 scores, it still exhibited limitations in managing nonlinear relationships between features.

Artificial Neural Networks (ANN), due to their strong nonlinear feature extraction capability, performed well when processing high-dimensional data. Its accuracy and F1 scores surpassed those of both Naive Bayes and Support Vector Machine. However, ANN's performance heavily depends on the network's depth and parameter tuning, and it requires longer training times.

XGBoost, a gradient boosting decision tree model, builds a strong classifier by enhancing the performance of weak classifiers, demonstrating significant advantages in handling complex data. XGBoost achieved high accuracy and F1 scores in our experiments, showcasing its strong performance in feature learning and classification.

The proposed CNN1D-Attention model first extracted local patterns from the time-series data of the physical fitness tests using a one-dimensional convolutional network (CNN1D). The attention mechanism further enhanced the model's feature selection ability, ensuring focus on the most important features. Experimental results showed that the CNN1D-Attention model performed exceptionally well in terms of accuracy and F1 score, significantly outperforming other traditional machine learning models.

Finally, the hybrid CNN1D-Attention and LightGBM model achieved the best performance. By inputting the deep features extracted by CNN1D-Attention into LightGBM for classification, the model fully leveraged the feature extraction capabilities of deep learning and the efficient classification power of LightGBM. This combination greatly improved both accuracy and F1 score, demonstrating the strong performance of the hybrid model in handling complex tasks.

Table 4 illustrates the experimental results of each model, highlighting the clear advantage of our proposed hybrid model in BMI classification tasks. These results indicate that combining deep learning with traditional machine learning provides better capture of complex feature relationships, leading to more accurate classification outcomes.

### 4.2 Limitations

This study has several limitations. First, it included only male university students due to the demographic composition of the participating institution, where female enrollment was too low to allow statistically meaningful analysis. As a result, the findings cannot be generalized to the entire student population.

Second, although multiple physical fitness indicators were used, the analysis did not further distinguish how each specific component (e.g., cardiorespiratory endurance, muscular strength, agility) individually correlates with other health outcomes, such as cognitive function or psychological status.

Third, the proposed model focused exclusively on BMI classification, without incorporating other health indicators such as metabolic risk or cardiovascular condition.

Lastly, the performance of the model may be influenced by the characteristics of the dataset used. As the data were collected from a single region and institution, caution should be exercised when applying the results to broader populations or different environments.

### 4.3 Practical implications and future directions

This study yields actionable insights for health governance bodies engaged in professional fitness management. By identifying cardiorespiratory endurance and upper-body strength as the most predictive physical indicators, our framework enables early detection of students at risk of developing suboptimal BMI categories. This capacity supports personalized exercise prescriptions and optimizes the allocation of health resources within higher educational institutions.

The proposed Hybrid Deep Learning Framework demonstrates significant translational potential for integration into mobile health (mHealth) applications and intelligent fitness platforms. When incorporated with appropriate interface architecture, it can provide real-time BMI assessments while dynamically adapting training recommendations based on individual performance trajectories.

Future investigations should incorporate multidimensional health determinants–including but not limited to dietary patterns, sleep metrics, and psychological wellbeing indicators–to enhance model robustness. Expansion of cohort diversity through inclusion of female participants and geographically distinct populations would substantially improve generalizability. Moreover, longitudinal designs capturing temporal dynamics in fitness-BMI relationships will facilitate the development of adaptive predictive models for continuous health surveillance.

## 5 Conclusion

The performance of this framework should be evaluated primarily through its translational utility rather than isolated statistical metrics. This methodology addresses fundamental limitations of conventional BMI assessment by enabling predictive trend analysis for predefined fitness objectives, thereby facilitating proactive weight regulation prior to clinical manifestation of health risks. It establishes quantifiable relationships between BMI modifications and physical performance enhancements—exemplified by observable improvements in endurance running outcomes following targeted BMI optimization—delivering evidence-based foundations for training regimen design.

When implemented in institutional contexts possessing requisite health monitoring infrastructure, the system demonstrates potential for reducing BMI surveillance expenditures relative to manual protocols. This operational efficiency is achieved while identifying critical physiological patterns—such as paradoxical co-occurrences of fitness gains and BMI escalation—to enable precision interventions. Furthermore, the translation of defined fitness milestones into physiologically congruent BMI parameters provides a mechanistic basis for next-generation AI-driven exercise prescription systems.

## Data Availability

The raw data supporting the conclusions of this article will be made available by the authors, without undue reservation.

## References

[1] Gill JMR Malkova D. Physical activity, fitness and cardiovascular disease risk in adults: interactions with insulin resistance and obesity. Clin Sci. (2006) 110:409–25. 10.1042/CS20050207 16526946

[2] Lavie CJ McAuley PA Church TS Milani RV Blair SN. Obesity and cardiovascular diseases. J Am Coll Cardiol. (2014) 63:1345–54. 10.1016/j.jacc.2014.01.022 24530666

[3] Oktay AA Lavie CJ Kokkinos PF Parto P Pandey A Ventura HO. The interaction of cardiorespiratory fitness with obesity and the obesity paradox in cardiovascular disease. Prog Cardiovasc Dis. (2017) 60:30–44. 10.1016/j.pcad.2017.05.005 28502849

[4] Kokkinos P Myers J. Exercise and physical activity: clinical outcomes and applications. Circulation. (2010) 122:1637–48. 10.1161/CIRCULATIONAHA.110.948349 20956238

[5] Sedeaud A Marc A Marck A Dor F Schipman J Dorsey M. BMI, a Performance parameter for speed improvement. PLoS ONE. (2014) 9:e90183. 10.1371/journal.pone.0090183 24587266 PMC3934974

[6] Zhou X Chen L Liu HX. Applications of machine learning models to predict and prevent obesity: a mini-review. Front Nutr. (2022) 9:933130. 10.3389/fnut.2022.933130 35866076 PMC9294383

[7] Smith BM Criminisi A Sorek N Harari Y Sood N Heymsfield SB. Modeling health risks using neural network ensembles. PLoS ONE. (2024) 19:e0308922. 10.1371/journal.pone.0308922 39383158 PMC11463747

[8] Cheng X Lin Sy Liu J Liu S Zhang J Nie P. Does physical activity predict obesity–a machine learning and statistical method-based analysis. Int J Environm Res Public Health. (2021) 18:3966. 10.3390/ijerph18083966 33918760 PMC8069304

[9] Jeon J Lee S Oh C. Age-specific risk factors for the prediction of obesity using a machine learning approach. Front Public Health. (2023) 10:998782. 10.3389/fpubh.2022.998782 36733276 PMC9887184

[10] Wang TY Cui J Fan Y. A wearable-based sports health monitoring system using CNN and LSTM with self-attentions. PLoS ONE. (2023) 18:e0292012. 10.1371/journal.pone.0292012 37819909 PMC10566674

[11] Júnior AC França AK Santos ED Silveira V Santos AD. Artificial neural networks to predict metabolic syndrome without invasive methods in adolescents. J Clini Med. (2024) 13:5914. 10.3390/jcm13195914 39407974 PMC11477488

[12] Yang H Yu B OUYang P Li X Lai X Zhang G. Machine learning-aided risk prediction for metabolic syndrome based on 3 years study. Sci Rep. (2022) 12:2248. 10.1038/s41598-022-06235-2 35145200 PMC8831522

[13] Mohammad F Al-Ahmadi S. WT-CNN: a hybrid machine learning model for heart disease prediction. Mathematics. (2023) 11:4681. 10.3390/math11224681

[14] Srinivasan S Francis D Mathivanan SK Rajadurai H Shivahare BD Shah MA. Hybrid deep CNN model for brain tumor image multi-classification. BMC Med Imaging. (2024) 24:21. 10.1186/s12880-024-01195-7 38243215 PMC10799524

[15] Madan P Singh V Chaudhari V Albagory Y Dumka A Singh R. An optimization-based diabetes prediction model using CNN and bi-directional LSTM in real-time environment. Appl Sci. (2022) 12:3989. 10.3390/app12083989

[16] Xiong X Wang A He J Wang C Liu R Sun Z. Application of lightGBM hybrid model based on TPE algorithm optimization in sleep apnea detection. Front Neurosci. (2024) 18:1324933. 10.3389/fnins.2024.1324933 38440395 PMC10909841

[17] Sasi D Joseph T Kanakambaran S. Hybrid CNN-lightGBM architecture for earthquake event classification in DAS systems. Arab J Sci Eng. (2025) 50:5573–87. 10.1007/s13369-024-09448-x

[18] Fang M Chen Y Xue R Wang H Chakraborty N Su T. A hybrid machine learning approach for hypertension risk prediction. Neural Comp Appl. (2023) 35:14487–97. 10.1007/s00521-021-06060-0

[19] Elyan E Moreno-Garcia CF Jayne C. CDSMOTE: class decomposition and synthetic minority class oversampling technique for imbalanced-data classification. Neural Comp Appl. (2021) 33:2839–51. 10.1007/s00521-020-05130-z

[20] Joloudari JH Marefat A Nematollahi MA Oyelere SS Hussain S. Effective class-imbalance learning based on SMOTE and convolutional neural networks. Appl Sci. (2023) 13:4006. 10.3390/app13064006

[21] Elagizi A Kachur S Carbone S Lavie CJ Blair SN. A review of obesity, physical activity, and cardiovascular disease. Curr Obes Rep. (2020) 9:571–81. 10.1007/s13679-020-00403-z 32870465

